# Supplementary material for: Association between complementary and alternative medicine use and prolonged time to conventional treatment among Thai cancer patients in a tertiary-care hospital
Source: PeerJ. 2019 Jun 14;7:e7159. doi: 10.7717/peerj.7159 (PMC6573806; doi:10.7717/peerj.7159)
Supplement: Supplemental Information 2 [file peerj-07-7159-s002.pdf]

Interview protocol form: Research project no:5/2018

Patient (Title and Name): ____

Interviewer: ____

Introduction:

1. Introduce yourself
2. Describe the study:
   "This survey is designed to assess the use of alternative therapy in this hospital. I have planned this interview to last no longer than half an hour. During this time, I have several questions that I would like to cover. All information will be held confidential. Your participation is voluntary and you may stop at any time if you feel uncomfortable."

Obtain the written informed consent:

1. Let the patient read the information sheet or read out to the patient.
2. Obtain signatures of the patient and relatives

Use of alternative therapy:

"Have you ever used treatments in the following list?"

1. traditional medicine
2. herbal medicine
3. diet supplements and vitamins
4. acupuncture
5. massage
6. meditation
7. any other therapy not provided by clinics and hospital

If yes, process with "Details of use" and "Patient-rated outcome"
If no, process with "General information"

Details of use:

1. "Can you describe the treatment? *"
   1.1 What is the name of treatment?
   1.2 How did the medicine use?
   1.3 Who provided it?
   1.4 Do you know the major component of the treatment?
2. "Can I look at the medicine?" (If yes, obtain information by direct examination of the product and skip to question no. 4.) *
3. "Does it have FDA registration?" If yes, is it registered as drug or diet supplement? *
4. "Did you use this medicine to heal cancer or alleviate cancer-related symptoms?" **
5. "Did you use this medicine after you became aware of the cancer diagnosis?" **
6. "How much the medicine cost?"
7. "Did you use the alternative treatment before receiving conventional treatments at Chonburi cancer hospital or other referring hospitals?"

* Classification of the herbal medicines and diet supplements is based on the presence or absence of registration with the Thai Food and Drug Administration. Fresh herbs, decoctions, infusions, and unauthorized commercial products are categorized as "homemade products".

** Patients will be categorized in the group that used alternative therapy if the alternative treatment was used with the intent to treat the cancer or was started after the patient became aware of the cancer diagnosis.

Explore: Why did the patient select the alternative treatment?
**Patient-rated outcome:**<br>Ask the patient to rate the satisfaction of using the alternative treatment with one of the following choices.<br>A. Good (i.e. the treatment is effective.)<br>B. Indifference (i.e. the treatment has no noticeable effect.)<br>C. Bad: (i.e. I had a bad experience with using the alternative medicine)<br><br>Explore: Why?
**General information:**<br>1. "How much was your average monthly income at the time before the diagnosis of cancer?"<br>2. "Did you ever leave out or delay your cancer treatment?"<br>If yes; Explore
**Finish the interview:**<br>"If you have any questions, you can ask now or later. If you wish to ask questions later, you may contact at phone number in the information sheet. Thanks so much for speaking with me today."

Data collection form

Name……………………………………………………………………………………………………… Age………………………….
Research number…………………………………………..Address…………………………………………………………….

Data from medical record review

Diagnosis…………………………………………………. Stage ☐I ☐ II ☐ III ☐ IV

Healthcare benefit scheme: ☐ Universal coverage ☐ Civil-servant ☐Social security ☐Self-pay

1. Date of biopsy procedure: ……../………. /………
2. Date of pathologic report: ……../………. /………
3. Referring date: ……../………. /………
4. Date of first attend to Cancer hospital: ……../………. /………
5. Date of first treatment ☐Surgery ☐ Chemotherapy ☐Radiation ☐ Chemo-Radiation
   Start: ……../………. /……… End: ……../………. /………
6. Date of second treatment ☐Surgery ☐ Chemotherapy ☐Radiation ☐ Chemo-Radiation
   Start: ……../………. /……… End: ……../………. /………
7. Date of third treatment ☐Surgery ☐ Chemotherapy ☐Radiation ☐ Chemo-Radiation
   Start: ……../………. /……… End: ……../………. /………

Wait time……………………….(day)
Note:

Loss to follow-up (if documented in medical record)
: ☐No
☐Yes ☐Before treatment ☐ During treatment

Use of alternative therapy:
☐No
☐Yes: Name………
Types: ☐Medication;
☐ Registered herbal medicine
☐ Diet supplement
☐ Homemade medicine
☐ Unknown
Ingredient……………………………………………………………………………………………………….
Intake and preparation…………………………………………………………………………………………….
☐ Non medication……..

Detail of treatment<br><br><br><br><br><br><br>☐Use before first conventional treatment<br>☐Use concurrent with conventional treatment<br>☐Use after conventional treatment<br><br>Cost………………………………………………..
Reason of use
Patient-rated outcome<br>☐Good ☐Indifference ☐ Bad<br>Why?
Average monthly income…………………………………………………………………
(if any) Reason why loss to follow up
