# Supplementary material for: Association between complementary and alternative medicine use and prolonged time to conventional treatment among Thai cancer patients in a tertiary-care hospital
Source: PeerJ. 2019 Jun 14;7:e7159. doi: 10.7717/peerj.7159 (PMC6573806; doi:10.7717/peerj.7159)
Supplement: Supplemental Information 3 — The picture is modified from the original map (https://d-maps.com/carte.php?num_car=3981&lang=en). [file peerj-07-7159-s003.pdf]

Supplementary contents

Sample size calculation

The target sample size for the present study was calculated using a formula based on an assumption that 40% of the patients would have used unconventional therapy. The desired precision was 0.05 with a 95% confidence interval. The minimum required sample size was estimated to be 369 patients, and we aimed to recruit at least 400 patients.

$n = Z^2_{1-\alpha/2}\, p\,(1-p)/d^2$

p = assumed prevalence = 0.4

Z = Z value for 95% confidence level) 95% CI = 1.96

d = desired precision = 0.05

n = Sample = 369

The study area map

The red area represents the nearby provinces and the blue area represents the distant provinces.

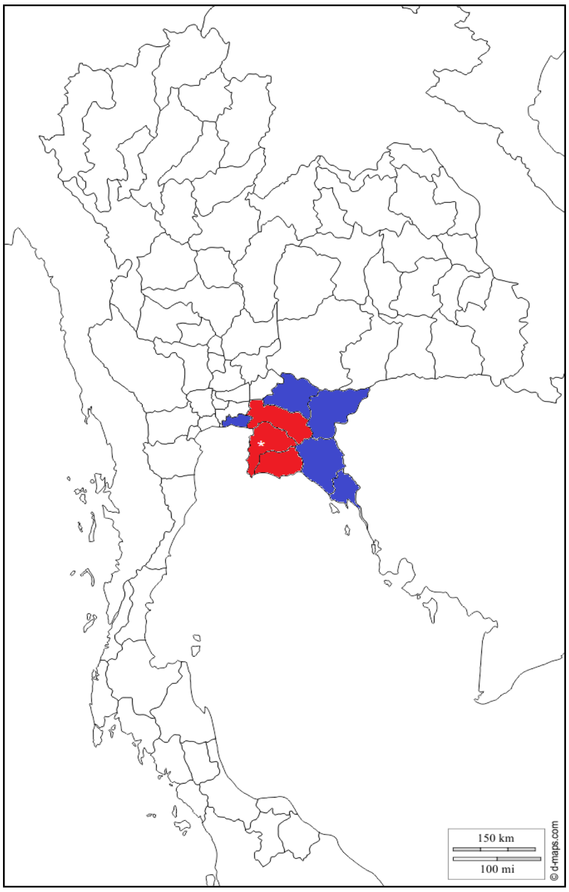

(Modified from the original map; https://d-maps.com/pays.php?num_pay=112&lang=en)
